# Constitutive IP_3_ signaling underlies the sensitivity of B-cell cancers to the Bcl-2/IP_3_ receptor disruptor BIRD-2

**DOI:** 10.1038/s41418-018-0142-3

**Published:** 2018-06-13

**Authors:** Mart Bittremieux, Rita M. La Rovere, Haidar Akl, Claudio Martines, Kirsten Welkenhuyzen, Kathia Dubron, Myriam Baes, Ann Janssens, Peter Vandenberghe, Luca Laurenti, Katja Rietdorf, Giampaolo Morciano, Paolo Pinton, Katsuhiko Mikoshiba, Martin D. Bootman, Dimitar G. Efremov, Humbert De Smedt, Jan B. Parys, Geert Bultynck

**Affiliations:** 1 Lab. Molecular and Cellular Signaling, Department of Cellular and Molecular Medicine and Leuven Kanker Instituut, KU Leuven, Leuven, Belgium; 2 Molecular Hematology Unit, ICGEB, Trieste, Italy; 3 Cell Metabolism, Department of Pharmaceutical and Pharmacological Sciences, KU Leuven, Leuven, Belgium; 4 Department of Hematology, UZ Leuven, Leuven, Belgium; 5 Department of Human Genetics, KU Leuven, Leuven, Belgium; 6 Università Cattolica del Sacro Cuore, Fondazione Policlinico A. Gemelli, Rome, Italy; 7 School of Life, Health and Chemical Sciences, The Open University, Milton Keynes, UK; 8 Department of Morphology, Surgery and Experimental Medicine, Section of Pathology, Oncology and Experimental Biology and LTTA center, University of Ferrara, Ferrara, Italy; 9 GVM Care & Research, Maria Pia Hospital, Torino, Italy; 10 GVM Care & Research, Maria Cecilia Hospital, Cotignola, Italy; 11 The Laboratory for Developmental Neurobiology, Brain Science Institute, RIKEN, Wako, Saitama Japan; 12 Present Address: Department of Biology, Lebanese University, Hadath, Lebanon

**Keywords:** Oncogenes, Calcium channels, Oncogene proteins

## Abstract

Anti-apoptotic Bcl-2 proteins are upregulated in different cancers, including diffuse large B-cell lymphoma (DLBCL) and chronic lymphocytic leukemia (CLL), enabling survival by inhibiting pro-apoptotic Bcl-2-family members and inositol 1,4,5-trisphosphate (IP_3_) receptor (IP_3_R)-mediated Ca^2+^-signaling. A peptide tool (Bcl-2/IP_3_R Disruptor-2; BIRD-2) was developed to abrogate the interaction of Bcl-2 with IP_3_Rs by targeting Bcl-2′s BH4 domain. BIRD-2 triggers cell death in primary CLL cells and in DLBCL cell lines. Particularly, DLBCL cells with high levels of IP_3_R2 were sensitive to BIRD-2. Here, we report that BIRD-2-induced cell death in DLBCL cells does not only depend on high IP_3_R2-expression levels, but also on constitutive IP_3_ signaling, downstream of the tonically active B-cell receptor. The basal Ca^2+^ level in SU-DHL-4 DLBCL cells was significantly elevated due to the constitutive IP_3_ production. This constitutive IP_3_ signaling fulfilled a pro-survival role, since inhibition of phospholipase C (PLC) using U73122 (2.5 µM) caused cell death in SU-DHL-4 cells. Milder inhibition of IP_3_ signaling using a lower U73122 concentration (1 µM) or expression of an IP_3_ sponge suppressed both BIRD-2-induced Ca^2+^ elevation and apoptosis in SU-DHL-4 cells. Basal PLC/IP_3_ signaling also fulfilled a pro-survival role in other DLBCL cell lines, including Karpas 422, RI-1 and SU-DHL-6 cells, whereas PLC inhibition protected these cells against BIRD-2-evoked apoptosis. Finally, U73122 treatment also suppressed BIRD-2-induced cell death in primary CLL, both in unsupported systems and in co-cultures with CD40L-expressing fibroblasts. Thus, constitutive IP_3_ signaling in lymphoma and leukemia cells is not only important for cancer cell survival, but also represents a vulnerability, rendering cancer cells dependent on Bcl-2 to limit IP_3_R activity. BIRD-2 seems to switch constitutive IP_3_ signaling from pro-survival into pro-death, presenting a plausible therapeutic strategy.

## Introduction

Different malignancies, including B-cell cancers such as diffuse large B-cell lymphoma (DLBCL), are characterized by overexpression of the anti-apoptotic Bcl-2 protein [1]. This proto-oncogene is localized at the mitochondria and at the endoplasmic reticulum (ER). At the level of the mitochondria, Bcl-2 binds to and neutralizes pro-apoptotic BH3-only proteins via its hydrophobic cleft, thereby preventing Bak/Bax activation and mitochondrial outer membrane permeabilization [2]. BH3-mimetic compounds, like venetoclax, counteract Bcl-2′s anti-apoptotic function at the mitochondria [3]. These molecules trigger apoptosis in cancer cells that are primed to death due to high levels of Bax or Bim, and thus are addicted to Bcl-2 for their survival [4, 5].

However, some cancer cells with high Bcl-2 levels respond poorly to BH3 mimetics [6–9], suggesting that Bcl-2 promotes cell survival via a different mechanism. Indeed, the last decades, Bcl-2 proteins emerged as critical modulators of intracellular Ca^2+^ dynamics [10, 11]. As such, Bcl-2 also acts at the ER Ca^2+^ stores where it inhibits inositol 1,4,5-trisphosphate (IP_3_) receptors (IP_3_Rs), a major class of intracellular Ca^2+^-release channels [12, 13]. Bcl-2 impacts IP_3_Rs by binding with its N-terminal BH4 domain to the central, modulatory domain of the channel [14–16]. Furthermore, Bcl-2′s C-terminal transmembrane domain enables efficient IP_3_R inhibition within cells [17]. A cell-permeable peptide tool named Bcl-2/IP_3_R Disruptor-2 (BIRD-2) was developed, capable of stripping Bcl-2 from IP_3_Rs [18]. In contrast, the BH3-mimetic Bcl-2 inhibitor venetoclax is not able to disrupt Bcl-2/IP_3_R complexes [19]. In chronic lymphocytic leukemia (CLL) and DLBCL, BIRD-2 triggered pro-apoptotic Ca^2+^-release events, while sparing normal peripheral mononuclear blood cells [18, 20].

In a collection of DLBCL cell lines, we previously identified IP_3_R2-expression levels as an important determinant underlying BIRD-2 sensitivity [20]. Here, we investigated whether IP_3_R2 levels are the only determinant that dictates the BIRD-2 sensitivity of B-cell cancers. Of note, IP_3_R2 is the IP_3_R isoform that displays the highest sensitivity to its ligand IP_3_ [21, 22]. Interestingly, B-cell cancers, including DLBCL and CLL, display constitutive B-cell receptor (BCR) signaling [23–25]. A cascade of signaling proteins becomes activated downstream of the BCR, including phospholipase C gamma 2 (PLCγ2), which hydrolyzes phosphatidylinositol 4,5-bisphosphate (PIP_2_) into IP_3_. We investigated whether constitutive PLCγ2/IP_3_ signaling occurs in B-cell cancer models and whether this contributes to survival and BIRD-2 sensitivity in DLBCL with elevated IP_3_R2-expression levels. Our results indicate that cancer cells are addicted to Bcl-2 acting at the ER Ca^2+^ stores to regulate IP_3_R-mediated Ca^2+^ release. We found that disrupting the Bcl-2/IP_3_R interaction with BIRD-2 switched Ca^2+^ signaling within cancer cells from pro-survival to pro-death, resulting in cancer cell death.

## Results

### IP_3_R2 expression is necessary but not sufficient for sensitivity to BIRD-2

**Fig. 1:**
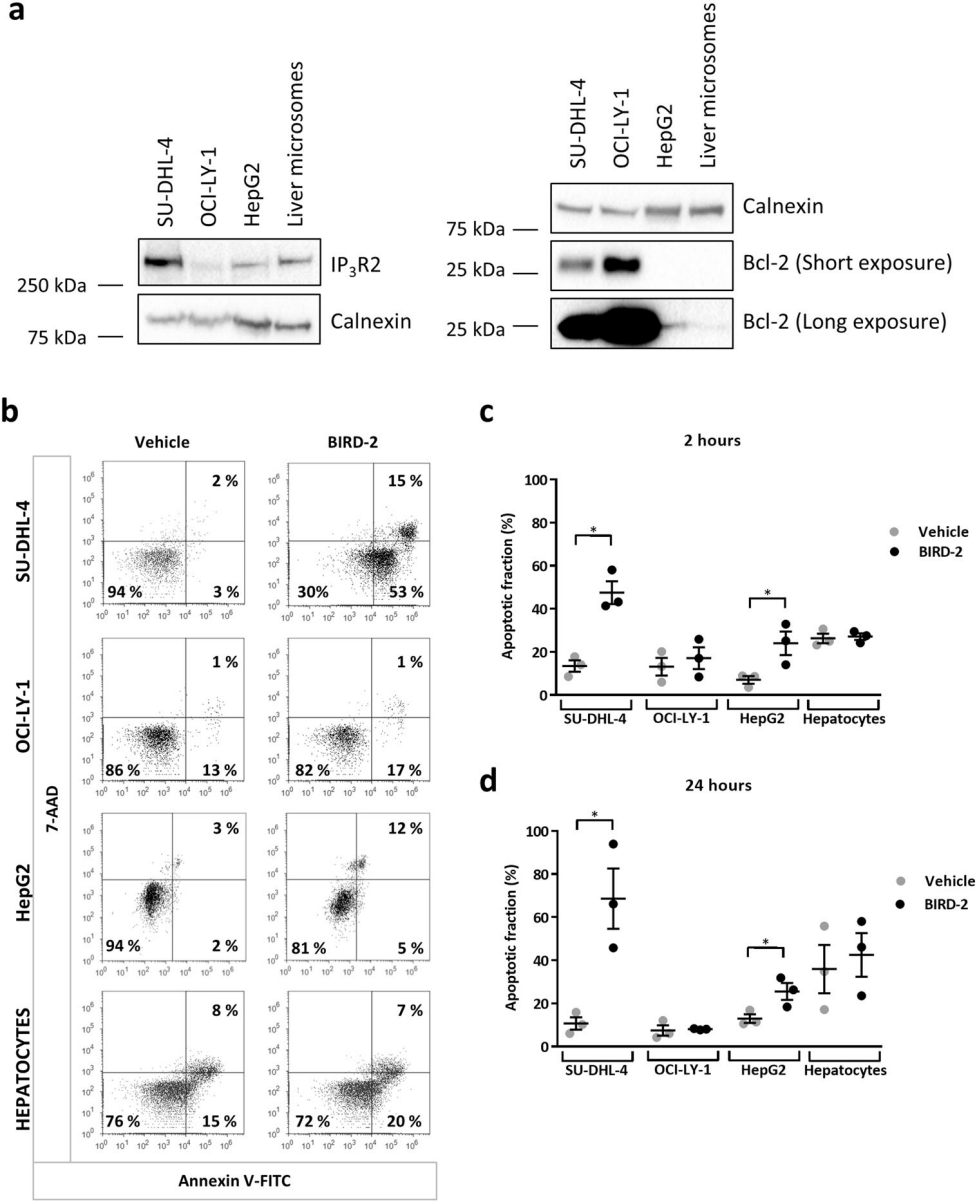
High IP_3_R2-expression levels are not sufficient *per se* to render cells sensitive to BIRD-2. **a** The IP_3_R2- and Bcl-2-protein levels present in cell lysates from SU-DHL-4 (40 µg), OCI-LY-1 (40 µg), and HepG2 (40 µg) cells and from microsomes extracted from primary hepatocytes (20 µg) were determined by western-blot analysis. The expression level of calnexin was used as a control for equal loading. **b** Representative dot plots from flow cytometry analysis measuring apoptosis by staining SU-DHL-4, OCI-LY-1, HepG2 cells and primary hepatocytes with Annexin V-FITC and 7-AAD. Cells were treated with vehicle or 10 μM BIRD-2 for 2 h. The dot plots are representative of 3 independent experiments. **c, d** Quantitative analysis of 3 independent experiments detecting apoptosis in Annexin V-FITC/7-AAD-stained cells treated with vehicle or 10 µM BIRD-2. Apoptotic cell death was measured 2 h (**c**) and 24 h (**d**) after BIRD-2 treatment. Data are represented as average ± SEM (*N* = 3). Statistically significant differences were determined with a Student’s *t*-test (paired, two-tailed, *P < 0.05) (BIRD-2 versus vehicle)

Since the sensitivity of DLBCL cell lines to BIRD-2 correlated to IP_3_R2-expression levels [20], we questioned whether IP_3_R2 expression is sufficient to dictate BIRD-2 sensitivity. Via western-blot analysis, we measured the expression levels of IP_3_R2 and Bcl-2 in microsomes prepared from primary hepatocytes, which have a high IP_3_R2 density [26–28], in human liver carcinoma HepG2 cells and in the BIRD-2-sensitive (SU-DHL-4) and BIRD-2-resistant (OCI-LY-1) DLBCL cell lines (Fig. 1a). This analysis revealed that IP_3_R2 is expressed in SU-DHL-4 and HepG2 cells, as well as in primary hepatocytes, while IP_3_R2 is virtually absent in OCI-LY-1 (Fig. 1a). Furthermore, the DLBCL cell lines expressed high levels of the anti-apoptotic Bcl-2 protein, whereas Bcl-2 expression was very low in the HepG2 cells or even absent in the liver microsomes (Fig. 1a). We next asked whether HepG2 cancer cells and primary hepatocytes are sensitive to BIRD-2. Therefore, apoptosis was measured in the four different cell types after 2 and 24 h of BIRD-2 (10 µM) treatment (Fig. 1b-d). BIRD-2 induced cell death in about 50% of the SU-DHL-4 cells, whereas OCI-LY-1 cells were not sensitive to 10 µM BIRD-2. In HepG2 cells, BIRD-2 induced apoptosis in approximately 20% of the population, suggesting that tumorigenic cells expressing IP_3_R2 display BIRD-2 sensitivity. To further substantiate the importance of IP_3_R2 for BIRD-2 sensitivity, primary hepatocytes were treated with 10 µM BIRD-2. Despite expression of IP_3_R2 (Fig. 1a), the hepatocytes were resistant to BIRD-2-induced apoptosis (Fig. 1b-d). Consistent with our previous findings [20], these data indicate that IP_3_R2 expression is required for BIRD-2-evoked apoptosis since tumorigenic cells lacking IP_3_R2 (OCI-LY-1) were resistant to BIRD-2, whilst tumorigenic cells expressing IP_3_R2 (SU-DHL-4) were sensitive. However, IP_3_R2 expression per se is not sufficient for BIRD-2-evoked cell death, since hepatocyte cell viability was not significantly affected by BIRD-2.

### SU-DHL-4 cells display enhanced basal IP_3_ signaling

**Fig. 2:**
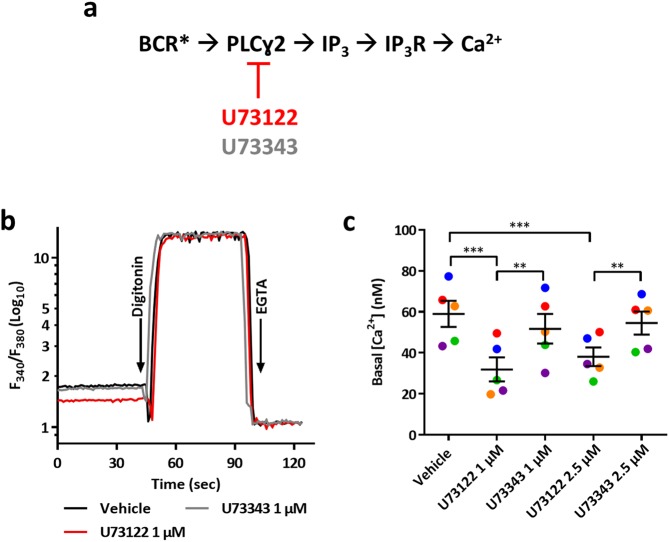
SU-DHL-4 cells display constitutive IP_3_/Ca^2+^ signaling. **a** The basal Ca^2+^ level was used as a read-out for measuring the level of constitutive IP_3_ signaling downstream the BCR, which has been reported to be tonically active in germinal center DLBCL (BCR*) [23–25]. PLC activity was suppressed using U73122, whereas its inactive enantiomer U73343 did not affect PLC activity. **b** A typical fluorescent recording of the basal [Ca^2+^]_cyt_ in SU-DHL-4 cells pre-treated with vehicle (black line), 1 μM U73122 (red line) or 1 µM U73343 (gray line) using the ratiometric Ca^2+^ indicator Fura-2 AM. The cells were present in Krebs medium supplemented with 1.5 mM CaCl_2_. The ratio of emitted fluorescence of Fura-2 (F_340_/F_380_) was monitored and Ca^2+^ values were calibrated by adding digitonin (50 µM) and a 20-fold excess of EGTA (33 mM) to determine *R*_max_ and *R*_min_ respectively (see Method section). Basal [Ca^2+^] (nM) are reported in **c** as the mean ± SEM (*N* = 5). The exact values of each independent experiment are represented in different colors. Statistically significant differences were determined using an analysis of variance (ANOVA, **P < 0.01, ***P < 0.001)

Since the BCR is tonically active in DLBCL cells and since the BCR signalosome activates PLCγ2 [23–25], we investigated whether the BIRD-2 sensitive SU-DHL-4 cell line displayed elevated constitutive IP_3_ signaling. As a read-out for constitutive IP_3_ signaling, we monitored the cytosolic Ca^2+^ concentration ([Ca^2+^]_cyt_) with Fura-2 in SU-DHL-4 cells pre-treated with vehicle, the PLC inhibitor U73122 or its inactive enantiomer U73343 (Fig. 2a-b). The basal [Ca^2+^]_cyt_ in vehicle-treated SU-DHL-4 cells was 59 ± 6.3 nM, which was lowered to 32 ± 5.6 and 38 ± 4.5 nM upon treatment with 1 and 2.5 μM U73122, respectively, while U73343 was without effect (Fig. 2c). Taken together, these results indicate that SU-DHL-4 cells are characterized by a constitutively active IP_3_ signaling, likely downstream to tonic BCR signaling.

### PLC inhibition suppresses BIRD-2-induced apoptosis in SU-DHL-4 cells

**Fig. 3:**
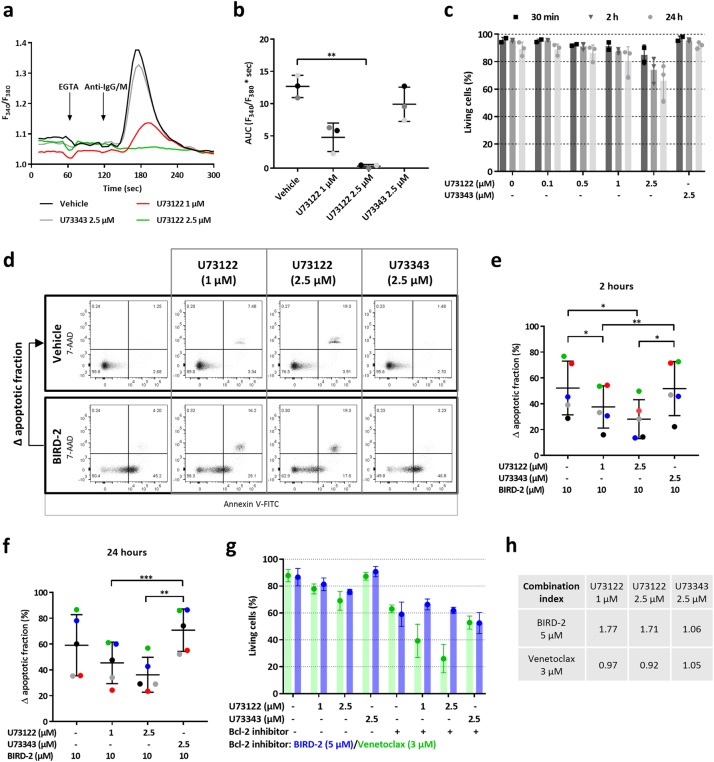
U73122 protects against BIRD-2-triggered apoptosis in SU-DHL-4. **a** Cell-population analysis of the cytosolic Ca^2+^ response, measured with Fura-2 AM, in SU-DHL-4 cells pre-treated for 30 min with U73122 (1 and 2.5 µM), U73343 (2.5 µM) or vehicle (DMSO). Addition of 3 mM EGTA and 12 µg/ml anti-IgG/M antibody is indicated by the first and second arrow, respectively. The curves are representative of 3 independent experiments. The cytosolic Ca^2+^ response after anti-IgG/M addition was quantified by measuring the area under the curve (AUC), which is shown in **b**. **c** Quantitative analysis of 3 independent experiments detecting apoptosis in Annexin V-FITC/7-AAD-stained SU-DHL-4 cells. Cells were treated with varying concentrations of U73122 or 2.5 µM U73343. Apoptotic cell death was measured 30 min, 2 h and 24 h after treatment. On the y-axis the percentage of living cells is plotted. Data are shown as the average ± SEM (*N* = 3). **d** Representative dot plots from flow cytometry analysis detecting apoptosis in Annexin V-FITC/7-AAD-stained SU-DHL-4 cells treated for 2 h with vehicle or 10 µM BIRD-2. Cells were pre-treated for 30 min with U73122 or U73343. **e, f** Quantitative analysis of 4 independent experiments detecting apoptosis in Annexin V-FITC/7-AAD-stained SU-DHL-4 cells. Apoptotic cell death was measured as the percentage of Annexin V-FITC-positive cells. Cells were pre-treated with U73122 or U73343 for 30 min. Cell death was measured 2 h (**e**) and 24 h (**f**) after BIRD-2 treatment. On the y-axis, the ∆ apoptotic fraction is plotted, which is the difference in apoptosis between the BIRD-2-treated and the vehicle-treated fraction, and between the BIRD-2 + U73122-treated and the U73122-treated fraction, and finally between the BIRD-2 + U73343-treated and the U73343-treated fraction. Data are shown as the average ± SEM (*N* = 5). **g** Quantitative analysis of 4 independent experiments detecting apoptosis in Annexin V-FITC/7-AAD-stained SU-DHL-4 cells treated with 1 or 2.5 µM U73122, 2.5 µM U73343, 5 µM BIRD-2 (blue), 3 µM venetoclax (green) or a combination of U73122/U73343 with BIRD-2/venetoclax. For the conditions without Bcl-2 inhibitor (indicated with a ‘-’), the green bars indicate the use of the vehicle control for venetoclax, while the blue bars indicate the use of vehicle control for BIRD-2 treatment. A ‘+’ indicates that the Bcl-2 inhibitor (BIRD-2/venetoclax) was added in this condition. Cell death was measured 24 h after treatment. On the y-axis the percentage of living cells, which corresponds to the Annexin V-FITC- and 7-AAD-negative fraction, is shown. Data are expressed as the average ± SEM (*N* = 4). **h** CI derived from SU-DHL-4 cells treated with U73122/U73343 in combination with BIRD-2/venetoclax. The CI was calculated (see Method section) from the data shown in **g**. Statistically significant differences were determined using an analysis of variance (ANOVA, *P < 0.05, **P < 0.01, ***P < 0.001)

To assess the contribution of IP_3_ signaling to BIRD-2-induced cell death, we blocked PLC signaling with U73122 in SU-DHL-4. In these cells, U73122 suppressed IP_3_-induced Ca^2+^ release, since the anti-IgG/M-provoked cytosolic Ca^2+^ response was reduced in cells pre-treated for 30 min with 1 and 2.5 µM U73122, compared to vehicle- or U73343-treated cells (Fig. 3a). This Ca^2+^ response was quantified by measuring the area under the curve (AUC), which was significantly reduced upon treatment with U73122 (2.5 µM) (Fig. 3b). Next, it was determined whether PLC inhibition by itself impacted SU-DHL-4 survival by treating them for 30 min, 2 h or 24 h with different concentrations of U73122 (0.1, 0.5, 1, and 2.5 µM) or U73343. Interestingly, the highest concentrations of U73122 (1 and 2.5 µM), but not its inactive enantiomer, induced apoptotic cell death in SU-DHL-4 cells (Fig. 3c). These data indicate that PLC signaling has a pro-survival role in DLBCL cells. Subsequently, it was determined whether PLC inhibition protected against BIRD-2-induced cell death in SU-DHL-4 cells. Therefore, apoptosis induced by 10 µM BIRD-2 was measured in cells pre-treated for 30 min with vehicle, U73122 or U73343 (Fig. 3d). After 2 h (Fig. 3e) and 24 h (Fig. 3f) of peptide treatment, U73122 significantly protected SU-DHL-4 cells against BIRD-2-triggered apoptosis, while U73343 did not. Of note, since U73122 provoked cell death by itself, the ∆ apoptotic fraction was calculated for each condition (Fig. 3e,f). The ∆ apoptotic fraction was obtained by subtracting the % of cells undergoing cell death in U73122-treated conditions from the % of cells undergoing cell death upon BIRD-2 + U73122 treatment (Fig. 3d).

To exclude that the protective effect of U73122 against BIRD-2-induced apoptosis was due to the artifact that less living cells were available for BIRD-2 upon U73122 treatment, we performed cell death assays in which SU-DHL-4 cells were treated with U73122 in combination with venetoclax, a Bcl-2-selective BH3-mimetic drug that provokes cell death independently of Ca^2+^ overload [19]. If U73122 and venetoclax work independently, the effect should be additive, providing a clear distinction with the data obtained with BIRD-2. After 24 h of venetoclax treatment, approximately 60% of the cells were alive (Fig. 3g). Combined treatment of venetoclax with U73122 (1 and 2.5 µM) further decreased the percentage of living cells compared to single treatment with venetoclax due to the cell death induced by U73122, while U73343 did not display this effect. In contrast, BIRD-2-induced cell death was decreased in combination with the PLC inhibitor, indicating that U73122 protected against BIRD-2-triggered apoptosis (Fig. 3g). Thus, the reduction in BIRD-2-induced cell death provoked by PLC inhibition is not due to a decreased availability of living cells upon U73122 treatment. To substantiate this further, we calculated the combination index (CI), which quantifies whether a drug combination is synergistic (CI < 0.8), additive (0.8 ≤ CI ≤ 1.2), or antagonistic (CI > 1.2) (Fig. 3h). The CI for the combined treatment of venetoclax with U73122 was approximately 1, indicating this drug combination is additive. In contrast, combined treatment of U73122 with BIRD-2 resulted in a CI of around 1.7, indicating an antagonistic drug combination. Hence, the ∆ apoptotic fraction provides a *bona fide* analysis for the protective effects of U73122 against BIRD-2-induced cell death (Fig. 3e,f).

Thus, these data indicate that PLC activity contributes to BIRD-2-induced DLBCL cancer cell death. This suggests that disrupting Bcl-2/IP_3_R complexes results in excessive, pro-apoptotic Ca^2+^ signals that are driven by endogenous IP_3_ signaling, whereby Bcl-2 suppresses such pro-death Ca^2+^ fluxes by tuning-down IP_3_R activity. Moreover, the increased basal PLC activity in DLBCL cells is a pro-survival signal, which can be changed to pro-death signaling by BIRD-2.

### PLC inhibition blunts the BIRD-2-induced cytosolic [Ca^2+^] rise in SU-DHL-4 cells

**Fig. 4:**
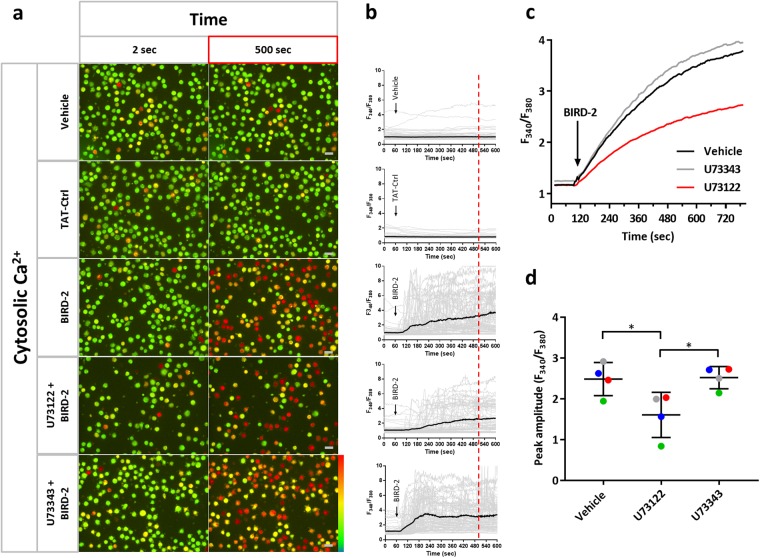
U73122 reduces the BIRD-2-induced cytosolic Ca^2+^ rise in SU-DHL-4 cells. **a** Single-cell analysis of the BIRD-2-induced Ca^2+^ response in SU-DHL-4 cells using the ratiometric Ca^2+^ indicator Fura-2 AM. Representative pseudo-color images before (2 s) and after (500 s) BIRD-2 treatment are shown. Vehicle and TAT-Ctrl were used as negative control conditions. The pseudo-color scale bar indicates increasing ratio fluorescence. **b** Single-cell cytosolic Ca^2+^ signals (gray lines) and their respective mean (black line) upon addition of vehicle, TAT-ctrl peptide or 10 µM BIRD-2 to SU-DHL-4 cells without or with pre-treatment of 1 μM U73122/U73343. **c** Cell-population analysis of the cytosolic Ca^2+^ response induced by 10 µM BIRD-2 in SU-DHL-4 cells pre-treated without (black line) or with 1 µM U73122 (green line) or 1 µM U73343 (gray line). The curves are representative of 4 independent experiments. Data were quantified by calculating the peak amplitude (**d**). In **d**, data are represented as mean ± SEM (*N* = 4). Statistically significant differences were determined using an analysis of variance (ANOVA, *P < 0.05)

Next, we investigated in more depth how PLC inhibition prevented the BIRD-2-evoked death of SU-DHL-4 cells. As reported previously [20], BIRD-2 caused an IP_3_R-dependent increase in cytosolic Ca^2+^ levels in SU-DHL-4 cells. Here, we assessed BIRD-2-induced Ca^2+^ elevations in Fura-2-loaded SU-DHL-4 cells in the presence of U73122 using single cell (Fig. 4a,b) and cell population (Fig. 4c,d) Ca^2+^ measurements. BIRD-2, but not a TAT-control peptide, caused a rise in the cytosolic Ca^2+^ levels in SU-DHL-4 single cells as measured by fluorescence microscopy. This Ca^2+^ rise was less prominent in cells pre-treated with 1 μM U73122, but not with U73343 (Fig. 4a,b). Similar findings were obtained in SU-DHL-4 cell populations analyzed using a FlexStation 3 microplate reader (Fig. 4c). The peak amplitude of the BIRD-2-evoked Ca^2+^ rise was significantly lower in SU-DHL-4 cells pre-treated with 1 µM U73122 compared to cells treated with vehicle or U73343 (Fig. 4d).

### Buffering intracellular IP_3_ suppresses BIRD-2-induced apoptosis in SU-DHL-4 cells

**Fig. 5:**
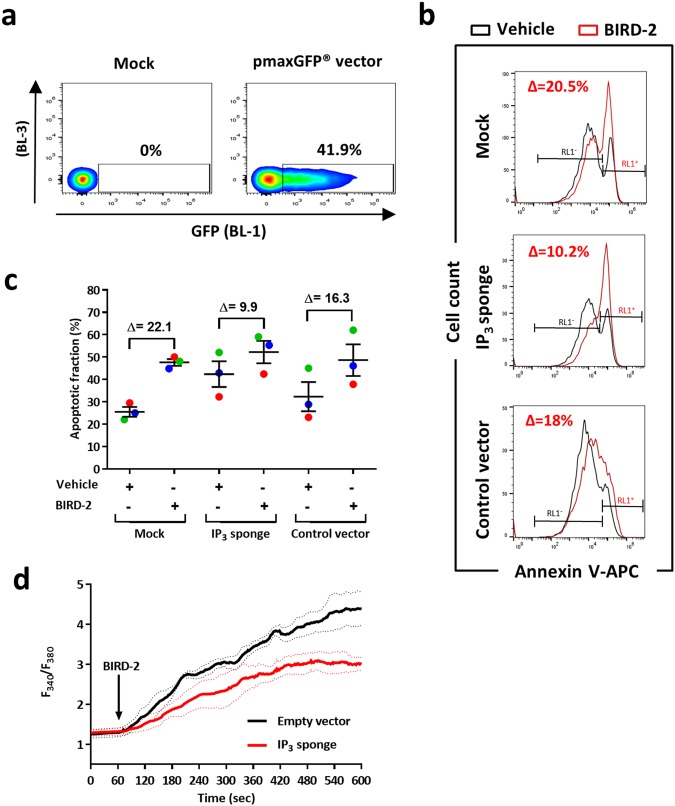
SU-DHL-4 cells are protected from BIRD-2-triggered apoptosis by genetically manipulating the IP_3_ signaling pathway. **a** Examples of flow cytometry analysis showing the percentage of GFP-positive transfected SU-DHL-4 cells, visible as a shift in the BL-1 (515–545 nm) channel, while the values in the BL-3 (665–715 nm) channel remained unaffected. **b** Representative flow cytometry analysis of BIRD-2-induced apoptosis in SU-DHL-4 cells transfected with the IP_3_ sponge or a control vector compared to mock-transfected cells. Apoptosis was detected via Annexin V-APC-positive staining (RL1^+^ = red laser, see Method section) of the cells. **c** Quantification of the apoptotic fraction (%) after treatment with 10 µM BIRD-2 (red histogram in panel **b**) or vehicle (black histogram panel **b**) in mock-transfected SU-DHL-4 cells or cells transfected with the IP_3_ sponge or a control vector. Apoptotic cells were identified as the Annexin V-APC-positive fraction (RL1^+^). Data are represented as mean ± SEM of 3 independent experiments. **d** Single-cell cytosolic Ca^2+^ measurements performed in SU-DHL-4 cells utilizing Fura-2 AM. Cells were transfected with an IP_3_ sponge vector or with an empty vector as negative control condition (pcDNA3.1). The addition of 10 µM BIRD-2 is indicated by the arrow. Data are represented as the average ± SEM of 3 independent experiments (*n* > 100 cells/condition)

Next, we aimed to confirm these findings by transfecting SU-DHL-4 cells with a high-affinity IP_3_ sponge that efficiently buffers intracellular IP_3_ [29]. Of note, approximately 40% of the SU-DHL-4 cells could be successfully transfected with our transfection method (Fig. 5a). BIRD-2-induced apoptosis was reduced in SU-DHL-4 cells expressing the IP_3_ sponge compared to mock-transfected or empty vector-transfected cells (Fig. 5b,c). The ∆ apoptotic fraction was approximately 22 and 16% in mock-transfected cells and SU-DHL-4 cells expressing a control vector, respectively (Fig. 5c). In contrast, the ∆ apoptotic fraction was only around 10% in SU-DHL-4 cells expressing the IP_3_ sponge. We also performed single-cell Ca^2+^ measurements, in which cells expressing the IP_3_ sponge displayed reduced BIRD-2-induced Ca^2+^ signals compared to empty vector-expressing cells (Fig. 5d). Hence, our pharmacological (U73122) and genetic (IP_3_ sponge) approaches indicate that constitutive IP_3_ signaling is an important determinant underlying BIRD-2 sensitivity in DLBCL. Moreover, the effect of the IP_3_ sponge demonstrates that IP_3_, rather than another messenger arising from upstream PLC activity, is critical for BIRD-2-evoked cell death.

### Pharmacological PLC inhibition also suppresses BIRD-2-induced apoptosis in other DLBCL cell lines

**Fig. 6:**
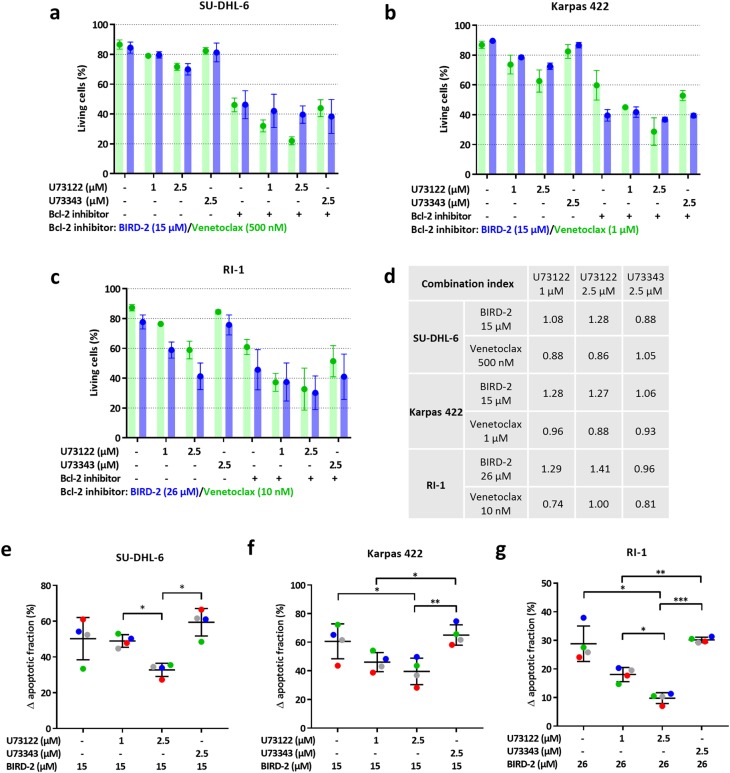
PLC inhibition also suppresses BIRD-2-triggered apoptosis in other DLBCL cell lines. **a**, **b**, **c** Quantitative analysis of at least 3 independent experiments detecting apoptosis in Annexin V-FITC/7-AAD-stained SU-DHL-6 (**a**), Karpas 422 (**b**), and RI-1 (**c**) cells treated with U73122, U73343, BIRD-2 (blue), venetoclax (green) or a combination of U73122/U73343 with BIRD-2/venetoclax. For the conditions without Bcl-2 inhibitor (indicated with a ‘-’), the green bars indicate the use of the vehicle control for venetoclax, while the blue bars indicate the use of vehicle control for BIRD-2 treatment. A ‘+’ indicates that the Bcl-2 inhibitor (BIRD-2/venetoclax) was added in this condition. SU-DHL-6 and Karpas 422 cells were treated with 15 µM BIRD-2, whereas 26 µM BIRD-2 was used to treat the RI-1 cells. SU-DHL-6 cells were treated with 500 nM venetoclax, Karpas 422 cells with 1 µM venetoclax and RI-1 cells were treated with 10 nM venetoclax. Cell death was measured 24 h after treatment. On the y-axis the percentage of living cells, which corresponds to the Annexin V-FITC- and 7-AAD-negative fraction, is shown. Data are expressed as the average ± SEM (*N* ≥ 3). **d** CI derived from cells treated with U73122/U73343 in combination with BIRD-2/venetoclax. The CI was calculated from the data shown in **a**, **b**, **c**. **e**, **f**, **g** Quantitative analysis of 4 independent experiments detecting apoptosis in Annexin V-FITC/7-AAD-stained SU-DHL-6 (**e**), Karpas 422 (**f**), and RI-1 (**g**) cells. Apoptotic cell death was measured as the percentage of Annexin V-FITC-positive cells. Cells were pre-treated with U73122 (1 or 2.5 µM) or U73343 (2.5 µM) for 30 min. Cell death was measured 24 h after BIRD-2 treatment. Data are shown as the ∆ apoptotic fraction, which is the difference in apoptosis between the BIRD-2-treated and the vehicle-treated fraction, and between the BIRD-2 + U73122-treated and the U73122-treated fraction, and finally between the BIRD-2 + U73343-treated and the U73343-treated fraction. Statistically significant differences were determined using an analysis of variance (ANOVA, *P < 0.05, **P < 0.01, ***P < 0.001)

It was examined whether constitutive IP_3_ signaling also contributes to BIRD-2-triggered apoptosis in other DLBCL cell lines besides SU-DHL-4, including Karpas 422 and SU-DHL-6 as germinal center DLBCL and RI-1, characterized as activated B-cell DLBCL. First, the U73122 sensitivity of these cells was determined by measuring apoptosis 24 h after treatment with U73122 (1 or 2.5 µM) or U73343 (2.5 µM) (Fig. 6a-c). U73122, but not the inactive enantiomer, induced cell death in the three cell lines. U73122-triggered apoptosis was the lowest in SU-DHL-6 (Fig. 6a), whereas RI-1 cells (Fig. 6c) were the most sensitive to PLC inhibition. These results indicate that all three DLBCL cell lines, like the SU-DHL-4 cells, depend on constitutive PLC/IP_3_ signaling for their survival. Next, it was investigated whether BIRD-2-induced apoptosis depends on this constitutive IP_3_ signaling (Fig. 6). In each cell line, the IC_50_ value of BIRD-2, previously determined in a subset of DLBCL [9], was used. This corresponds to 15 µM BIRD-2 for SU-DHL-6 (Fig. 6a-e) and Karpas 422 cells (Fig. 6b-f), and 26 µM for RI-1 (Fig. 6c-g). To determine U73122-mediated protection against BIRD-2 in these cell lines, the ∆ apoptotic fraction was used. This analysis was again validated using venetoclax (Fig. 6a-d) as before (Fig. 3g-h), showing that U73122 treatment displayed additive cell-death effects with venetoclax, whereas U73122 + BIRD-2 is an antagonistic drug combination (Fig. 6d). In all three cell lines tested, BIRD-2-induced cell death was significantly suppressed by U73122 (Fig. 6e-g). In conclusion, these data indicate that not only SU-DHL-4 but also other DLBCL cell lines depend on constitutive IP_3_ signaling for their survival, and that this pro-survival signaling can be turned into pro-death signaling by BIRD-2.

### Pharmacological PLC inhibition suppresses BIRD-2-induced apoptosis in primary CLL patient cells

**Fig. 7:**
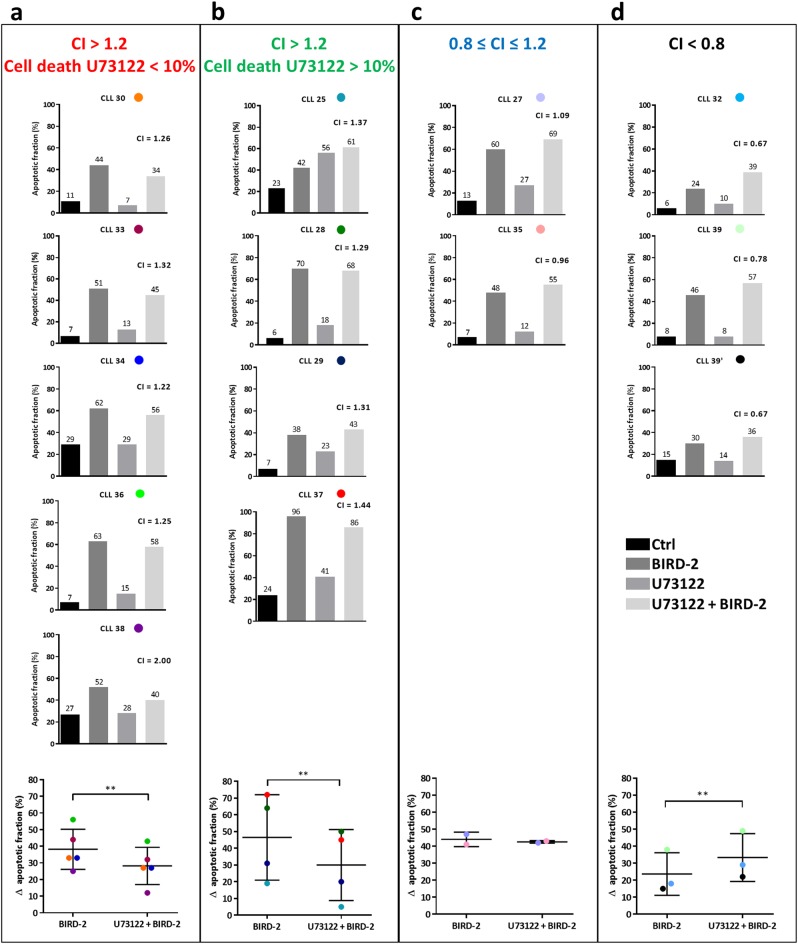
PLC inhibition suppresses BIRD-2-induced cell death in a subset of primary CLL patient cells. (**a**-**d**) Results from flow cytometry analysis of Annexin V-FITC/7-AAD-stained CLL patient samples treated for 2 h with 30 µM BIRD-2 with or without U73122 pre-treatment. For each CLL sample, the individual bar graph, plotting the apoptotic fraction (%) measured in untreated cells (black bar), cells treated with BIRD-2 (dark gray bar), U73122 (gray bar) or a combination of U73122 and BIRD-2 (light gray bar), is shown. The lowest U73122 concentration for which an effect could be observed was used (0.1/0.5/2.5 µM; see Supplemental Table 1). The CLL samples were stratified in 4 categories, according to the CI calculated for the combined treatment of U73122 and BIRD-2 and according to their sensitivity towards U73122: (**a**) CI > 1.2 & cell death U73122 < 10%; (**b**) CI > 1.2 and cell death U73122 > 10%; (**c**) 0.8 ≤ CI ≤ 1.2; (**d**) CI < 0.8. At the bottom of each panel, the ∆ apoptotic fraction (%), which corresponds to the difference in apoptotic fraction between the BIRD-2-treated and the control condition, and between the BIRD-2 + U73122-treated and the U73122-treated conditions, is shown for each CLL sample belonging to that category. In the dot plots, each CLL sample is represented with a different color, which is shown in the titles of the individual bar graphs. Statistically significant differences were determined using a one-tailed paired *t*-test (**P < 0.01)

Finally, we aimed to translate our findings to primary peripheral blood cells obtained from patients diagnosed with CLL, another B-cell malignancy characterized by constitutively active BCR signaling. BIRD-2 (30 µM) treatment for 2 h triggered apoptosis in all 14 CLL samples analyzed, though with potencies ranging from ~20% to ~70% of the cells being apoptotic (Fig. 7). To assess whether IP_3_ signaling contributes to the BIRD-2 response in the CLL cells, BIRD-2-triggered apoptosis was measured in samples pre-treated for 30 min with U73122. The lowest U73122 concentration for which an effect could be observed was used in each sample (0.1/0.5/2.5 µM, Supplemental Table 1). The CLL patient samples were stratified in groups according to their sensitivity towards U73122 and to the CI calculated for the combined treatment of U73122 with BIRD-2. In this way, four different groups are recognized: CI > 1.2 and cell death U73122 < 10% (Fig. 7a), CI > 1.2 and cell death U73122 > 10% (Fig. 7b), 0.8 ≤ CI ≤ 1.2 (Fig. 7c), and CI < 0.8 (Fig. 7d). In 9 out of 14 CLL samples, we found that the drug combination was antagonistic (CI > 1.2), suggesting that PLC inhibition protected against BIRD-2-induced apoptosis. However, because the CLL cells displayed varying sensitivity to U73122, the samples were further subdivided according to U73122-induced cell death. U73122 did not induce apoptosis in 5 of these samples (Fig. 7a), whereas cell viability of the other 4 samples was reduced by PLC inhibition (Fig. 7b). To determine whether U73122 significantly protected against BIRD-2-induced apoptosis in these groups, the ∆ apoptotic fraction for BIRD-2-treated and U73122 + BIRD-2-treated cells was calculated (Supplemental Table 1). This analysis indicates that U73122 significantly protected against BIRD-2-induced apoptosis in both groups (Fig. 7a-b). Next, in 2 out of 14 CLL samples, U73122 did not protect against BIRD-2 (0.8 ≤ CI ≤ 1.2) (Fig. 7c), whereas in 3 out of 14 samples BIRD-2-induced apoptosis was even increased by U73122 (CI < 0.8) (Fig. 7d). The ∆ apoptotic fraction analysis was also validated for these CLL samples using venetoclax (Supplemental Fig. 1).

**Fig. 8:**
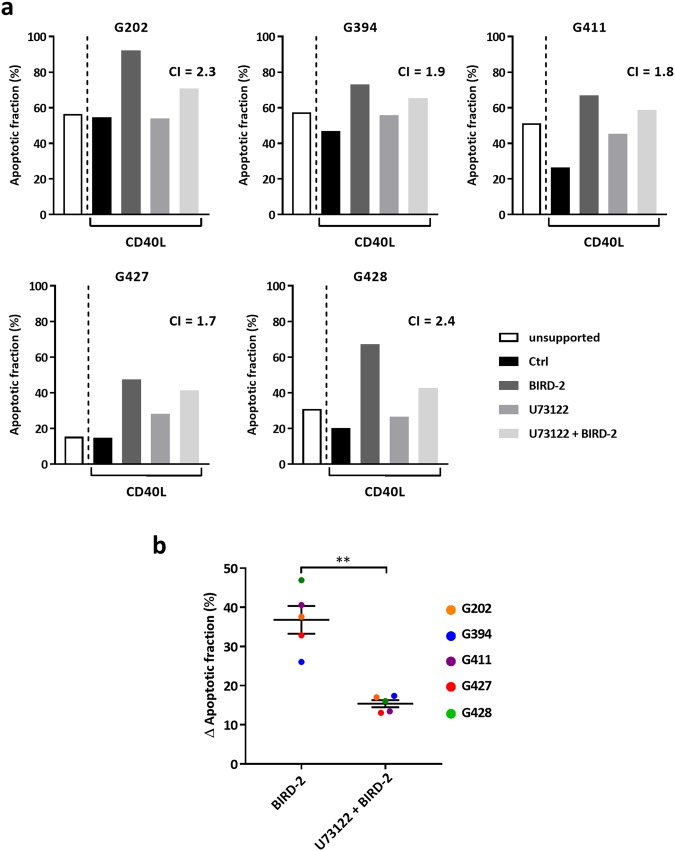
Pharmacological PLC inhibition protects against BIRD-2-induced apoptosis in co-cultured CLL cells. **a** Results from flow cytometry analysis of Annexin V-FITC/PI-stained CLL samples that were either unsupported or co-cultured with CD40L-expressing fibroblasts. The co-cultured CLL cells were treated for 20 h with vehicle or 30 µM BIRD-2 with or without 0.1 µM U73122 pre-treatment. Data are shown as the percentage of apoptotic cells (%). For each CLL sample, the CI calculated for U73122 + BIRD-2 treatment is indicated. **b** Plot of the ∆ apoptotic fraction (%) for BIRD-2 and U73122 + BIRD-2 treatment of each CLL sample in co-cultured conditions. The ∆ apoptotic fraction corresponds to the difference in apoptotic fraction between the BIRD-2-treated and the control condition, and between the BIRD-2 + U73122-treated and the U73122-treated conditions. Statistically significant differences were determined using a one-tailed paired *t*-test (**P < 0.01)

Finally, we measured the effect of U73122 on the BIRD-2 response in CLL cells co-cultured with CD40L-expressing fibroblasts to allow for longer BIRD-2 treatments. Co-cultured CLL cells were treated for 20 h with BIRD-2 and/or U73122, after which the cells were collected and cell viability was assessed. Co-cultured CLL cells appeared better protected from spontaneous apoptosis than CLL cells in non-supported cultures (Fig. 8a). In these co-cultured CLL cells, BIRD-2 remained capable to induce cell death and PLC inhibitor U73122 significantly reduced BIRD-2-induced apoptosis (Fig. 8b). Thus, CLL cells in both unsupported and supported cultures share a common sensitivity to BIRD-2, as disruption of the Bcl-2/IP_3_R interaction leads to death of the primary cells. Furthermore, in most CLL patient samples suppressing basal PLC activity with U73122 protected against BIRD-2-induced apoptosis. However, for some CLL cells, enhanced basal PLC signaling is very critical for their survival, and solely inhibiting PLC is sufficient to cause cell death.

## Discussion

The main finding of this study is that constitutive IP_3_ signaling, besides high IP_3_R2-expression levels, is an important determinant that underlies cancer cells’ addiction to Bcl-2 at the ER Ca^2+^ stores. Constitutive IP_3_ signaling is therefore an additional determinant of the sensitivity of B-cell cancers, like DLBCL and CLL, to BIRD-2, a Bcl-2 inhibitor that targets its BH4 domain and alleviates Bcl-2′s inhibitory role on IP_3_R channels. As such, BIRD-2 can be applied as a tool to exploit pro-survival constitutive IP_3_ signaling occurring in B-cell cancers and switch it into pro-apoptotic signaling.

BIRD-2 disrupts endogenous Bcl-2/IP_3_R complexes, thereby triggering Ca^2+^-driven apoptosis in different malignancies, including CLL [18, 20, 30], DLBCL [20], multiple myeloma [31], follicular lymphoma [31], and small cell lung cell carcinoma [32]. DLBCL cells displayed a varying sensitivity to BIRD-2, which correlated to the expression levels of IP_3_R2 [20]. Furthermore, a reciprocal sensitivity between BIRD-2 and venetoclax has been reported for DLBCL cells, indicating that cancer cells less sensitive to BH3 mimetics are more sensitive to BIRD-2 and vice versa [9]. Additionally, low BIRD-2 concentrations sensitized DLBCL cells towards venetoclax by upregulating the pro-apoptotic BH3-only protein Bim [9]. BIRD-2 also sensitized multiple myeloma cells to BH3 mimetics through a mechanism that involved the Ca^2+^-dependent upregulation of Bim [31]. In this study, we also measured Bim expression in SU-DHL-4 cells treated with higher concentrations of BIRD-2 (Supplemental Fig. 2). After 24 h of treatment with the IC_50_ value of BIRD-2 (10 µM), we observed a significant increase in Bim expression, suggesting that this BH3-only protein may contribute to BIRD-2-triggered cytotoxicity. However, further work is needed to elucidate the role of Bim in BIRD-2-induced apoptosis.

It is well established that DLBCL and CLL cells display chronic BCR signaling, leading to constitutive activation of different signaling pathways, including the PLCγ2 pathway, which leads to the production of IP_3_ in basal conditions [23–25]. We here show that IP_3_ levels are likely elevated in SU-DHL-4 cells, as a PLC inhibitor was able to lower basal [Ca^2+^]. We also attempted to directly measure IP_3_ levels using an IP_3_ FRET sensor [33], but the dynamic range of this sensor appeared insufficient to reliably assess a decrease in basal IP_3_ levels using our microscopy systems. The role of constitutive IP_3_ signaling in DLBCL cell survival requires further study, since pharmacological PLC inhibition using U73122 affected the survival of several DLBCL cell lines. We anticipate that this is an on-target effect of U73122 on PLC, since its inactive enantiomer U73433 did not display this effect. These findings indicate a pro-survival role of basal IP_3_/Ca^2+^ signaling, but further work is needed to document this in other B-cell cancers and lymphoproliferative malignancies. Nevertheless, these results converge with findings obtained in solid tumors, showing that tumorigenic, but not non-tumorigenic, cells depend on basal IP_3_R function for their survival [34–36]. In these cancer models, IP_3_Rs provide a constitutive ER-mitochondrial Ca^2+^ flux to drive mitochondrial metabolism and the production of mitochondrial substrates needed for nucleotide synthesis critical for cancer cell proliferation. Normal cells are less dependent on IP_3_Rs for their survival, as they can tune down proliferation to accommodate the compromised mitochondrial bio-energetics [34–36]. However, further research is needed to determine whether constitutive IP_3_ signaling and basal IP_3_R function are both essential for B-cell cancer cell survival by mediating ER-mitochondrial Ca^2+^ fluxes that sustain mitochondrial metabolism, thereby accounting for U73122-induced cell death [34, 35].

An important implication of this study is that although IP_3_R2 expression is important for BIRD-2-induced apoptosis, it is not sufficient *per se*. It is clear that a constitutively increased level of IP_3_, the ligand that activates IP_3_R channels, is needed as well. Of interest, IP_3_R2 channels display the highest IP_3_ sensitivity [22]_._ Thus, the combination of high IP_3_R2-expression levels and constitutive IP_3_ signaling makes DLBCL cells particularly addicted to Bcl-2 inhibition of IP_3_Rs at the ER, and thus sensitive to BIRD-2. This is supported by data obtained in primary hepatocytes, cells expressing relatively high levels of IP_3_R2, but which are resistant to BIRD-2, suggesting that IP_3_R2 alone is not sufficient for BIRD-2 sensitivity. These data are of high importance, as IP_3_R2 channels are expressed in different organs and tissues in the human body, where they exert important physiological functions [22]. Our data therefore suggest that BIRD-2-derived or BIRD-2-mimetic molecules may be well tolerated in the human body and may not cause a general toxicity in normal cells or tissues that express high IP_3_R2 levels.

The concept of constitutive IP_3_ signaling contributing to BIRD-2 sensitivity was also observed in primary CLL samples, where low concentrations of U73122 protected against BIRD-2-induced apoptosis. In the first place, we focused on the cell-autonomous response of the CLL cells towards BIRD-2. However, interactions with bystander cells in micro-environmental niches support CLL cells by providing survival and proliferative signals [37–39]. Hence, these unsupported experiments were restricted towards short-term BIRD-2 application to limit spontaneous cell death, correlating with loss of Bcl-2-family members such as anti-apoptotic Mcl-1 due to rapid loss of supportive signals [37]. Therefore, we also performed experiments in CLL cells supported by CD40L-expressing fibroblasts. These co-culture conditions protected against spontaneous apoptosis, but did not antagonize BIRD-2-induced cell death. Furthermore, PLC inhibition with U73122 remained capable of suppressing BIRD-2-induced apoptosis in CLL cells irrespective of whether they were exposed to BIRD-2/U73122 in unsupported or co-cultured conditions.

We observed variability in the sensitivity of individual CLL samples to BIRD-2, though the underlying mechanisms remain elusive. The BIRD-2 sensitivity of CLL cells did not correlate to their BCR mutational status, suggesting that basal IP_3_ signaling might be increased in B-cell cancers irrespective of their BCR mutational status (Supplemental Table 1). The varying BIRD-2 sensitivity could be due to differences in IP_3_R2 expression, due to varying deficiencies in regulators of IP_3_R function [40], or due to different degrees of coupling between the ER and the mitochondria [41]. For instance, phosphatase and tensin homolog (PTEN) and protein kinase B (Akt/PKB) control Ca^2+^-dependent apoptosis via IP_3_R3 [42–44]. Since reduced PTEN levels have been reported in CLL, as well as in DLBCL [45, 46], we measured PTEN expression in the primary CLL samples and DLBCL cell lines used in this study. All CLL samples expressed PTEN at similar levels (Supplemental Fig. 3a), indicating that differences in PTEN expression do not account for the varying BIRD-2 sensitivity of CLL cells. Furthermore, PTEN was detected in SU-DHL-4, SU-DHL-6 and RI-1 cells, but not in Karpas 422 (Supplemental Fig. 3b). Thus, BIRD-2 sensitivity of DLBCL cells appears unrelated to PTEN expression, since both PTEN-proficient SU-DHL-4 and PTEN-deficient Karpas 422 cells respond well to BIRD-2 (IC_50_ values around 10 µM) [9].

Overall, our study indicates that constitutive IP_3_ signaling, likely a pro-survival mechanism in B-cell malignancies, is an important contributor for BIRD-2-induced apoptosis in cancer cells that express high IP_3_R2 levels. Although IP_3_R2 is important for BIRD-2-induced cell death, its high expression alone is not sufficient *per se* for BIRD-2 sensitivity. This is important given the pivotal physiological functions of IP_3_R2 channels in normal tissues and cells. Hence, Bcl-2 antagonism via the BH4 domain might be a promising strategy to target B-cell cancers, in particular those displaying high IP_3_R2-expression levels and constitutive IP_3_ signaling.

## Materials and methods

### Reagents, antibodies, and constructs

Reagents were as follows: ethylene glycol tetraacetic acid (EGTA) (Acros Organics, Geel, Belgium, 409910250), Fura-2 AM (Biotium, Kampenhout, Belgium, 50033), Annexin V-Fluorescein isothiocyanate (FITC) (Becton Dickinson, Franklin Lakes, NJ, USA, 556419), 7-aminoactinomycin D (7-AAD) (Becton Dickinson, 555815), U73122 (Enzo Life Sciences, Farmingdale, NY, USA, BML-ST391-0005), U73343 (Enzo Life Sciences, BML-ST392-0005), venetoclax (ChemieTek, Indianapolis, IN, USA, CT-A199), anti-human IgG/M (Jackson ImmunoResearch, West Grove, PA, USA, 109-006-127). The following antibodies were used: anti-IP_3_R2 (Abiocode, Agoura Hills, CA, USA, R2872-3); anti-calnexin (Enzo Life Sciences, Farmingdale, NY, USA, ADI-SPA-865-D); anti-Bcl-2 (Santa Cruz Biotechnology, Dallas, TX, USA, sc7382HRP); anti-Bim (Bioké, Leiden, The Netherlands, 2819 S); anti-GAPDH (Sigma-Aldrich, St. Louis, MO, USA, G8795); anti-vinculin (Sigma-Aldrich, Munich, Germany, V9131). The sequences of the peptides used in this study were: BIRD-2 (RKKRRQRRRGGNVYTEIKCNSLLPLAAIVRV) and TAT-Ctrl (RKKRRQRRRGGSIELDDPRPR). These peptides were synthesized by LifeTein (South Plainfield, New Jersey, USA) with a purity of at least 85%. The IP_3_ sponge (pEF-GSTm49-IRES-GFP) is a protein constructed from the IP_3_-binding core of the type 1 IP_3_R with a single amino acid substitution (R441Q) that has a very high affinity for IP_3_ [29].

### CLL patient samples

CLL was defined by clinical examination of the patients and immunophenotypic analysis of the blood samples. Only samples where > 80% of the cells were CD19 + were considered. The tumor immunoglobulin heavy chain variable (IGHV) sequence was determined to designate the BCR status (unmutated or mutated). The collection of blood samples from CLL patients has been approved by the ethical committee of the UZ Leuven (Belgian Number: B3222001536) and by the ethical committee of the Università Cattolica del Sacro Cuore, Fondazione Policlinico A. Gemelli, Rome, Italy (protocol number 14563/15). Blood samples were collected according to the principles established by the International Conference on Harmonization Guidelines on Good Clinical Practice. An informed consent was obtained from all patients. Primary lymphocytes were separated using a Ficoll Hypaque density gradient from the peripheral blood of adult patients with B-CLL, and re-suspended in RPMI-1640 medium. For co-culture experiments, human CLL cells (1 × 10^7^/ml) were cultured for 24 h in the presence of CD40L-expressing fibroblasts (2 × 10^4^/condition), which were previously treated for 2 h with 10 µg/ml mitomycin C. The CLL co-cultures were then pre-treated for 90 min with U73122 prior to the addition of BIRD-2. After 20 h of BIRD-2 treatment, CLL cells were collected and analyzed.

### Cell culture

The SU-DHL-4, OCI-LY-1, Karpas 422, and SU-DHL-6 DLBCL cell lines were kindly obtained from Dr. Anthony Letai (Dana-Farber Cancer Institute, Boston, Massachusetts, USA). The RI-1 DLBCL cell line was obtained from DSMZ (Braunschweig, Germany). All these cell lines were authenticated by the University of Arizona Genetics Core (Tucson, AZ, USA) using autosomal short tandem repeat (STR) profiling utilizing the Science Exchange platform (www.scienceexchange.com). All cell lines fully matched the DNA fingerprint present in reference databases, except for SU-DHL-6 cells, which matched 7 out of 8 tested alleles. OCI-LY-1 cells were cultured in suspension in Iscove’s modified Dulbecco’s medium (Invitrogen, Merelbeke, Belgium), while the other DLBCL cell lines were cultured in suspension in RPMI-1640 medium (Invitrogen, Merelbeke, Belgium). The human hepatocellular carcinoma cell line HepG2 was cultured in Dulbecco’s Modified Eagle medium (DMEM). Media were supplemented with 10% heat-inactivated fetal bovine serum, L-glutamine (100 × GlutaMAX, Gibco/Invitrogen, 35050) and penicillin and streptomycin (100 × Pen/Strep, Gibco/Invitrogen, 15070-063). Cells were cultured at 37 °C in the presence of 5% CO_2_. Primary hepatocytes were isolated from mice using a two-step collagenase perfusion as previously described [47]. Subsequently, the primary cells were cultured in DMEM supplemented with 10% heat-inactivated fetal bovine serum, L-glutamine, penicillin and streptomycin.

### Cell transfection

Twenty-four hours after seeding, the indicated vectors were introduced into the SU-DHL-4 cells utilizing the Amaxa^®^ Cell Line Nucleofector^®^ Kit L (Lonza, Basel, Switzerland), program C-05. Briefly, 3 × 10^6^ cells were transfected with 3 μg of pEF-GSTm49-IRES-GFP (IP_3_ sponge vector), 3 μg pcDNA3.1 (control vector), or 1.5 μg pmaxGFP^®^ vector (to assess transfection efficiency). GFP expression was checked by flow cytometry 24 h after transfection. A pcDNA 3.1(-) mCherry expressing vector was co-transfected at a 1:3 ratio as a selection marker for single-cell cytosolic Ca^2+^ imaging.

### Apoptosis assay

DLBCL cells (5 × 10^5^ cells/ml) were treated as indicated, pelleted by centrifugation, and incubated with Annexin V-FITC/7-AAD or Annexin V-APC. Cell suspensions were analyzed with an Attune^®^ Acoustic Focusing Flow Cytometer (Applied Biosystems). Cell death by apoptosis was scored by quantifying the population of Annexin V-FITC-positive cells (blue laser; BL-1) or Annexin V-APC-positive cells (red laser; RL-1). The latter was used in combination with pEF-GSTm49-IRES-GFP. To assess the effect of U73122 on BIRD-2-induced cell death, the ∆ apoptotic fraction was obtained by subtracting the % of cells undergoing cell death in U73122-treated conditions from the % of cells undergoing cell death upon BIRD-2 + U73122 treatment. Flow-cytometric data were plotted and analyzed using Attune version 2.1.0 (Applied Biosystems) or FlowJo version 10 software. The CI was calculated in order to determine mathematically whether a drug combination is synergistic (CI < 0.8), additive (0.8 ≤ CI ≤ 1.2), or antagonistic (CI > 1.2). The CI was determined by making the ratio of the sum of the individual effects (Effect_Compound A_ + Effect_Compound B_) with the effect of the combined treatment (Effect_Compound A + Compound B_).

### Western-blot analysis

Cells were washed with phosphate-buffered saline and incubated at 4 °C with lysis buffer (25 mM HEPES, pH 7.5, 1% Triton X-100, 300 mM NaCl, 1.5 mM MgCl_2_, 10% glycerol, 2 mM EDTA, 2 mM EGTA, 1 mM dithiothreitol, and protease inhibitor tablets (Roche, Basel, Switzerland)) for 30 min on a head-over-head rotor. Cell lysates were centrifuged for 5 min at 10,000 r.p.m. and analyzed by western blotting as previously described [16]. Microsomes were prepared from primary hepatocyte as previously described [48].

### Basal [Ca^2+^]_cyt_ measurements

Basal Ca^2+^ levels were monitored with the cytosolic Ca^2+^ indicator Fura-2 AM. Cells (10 × 10^6^/sample) were loaded for 30 min with 1.25 µM Fura-2 AM at room temperature in modified Krebs solution (containing 150 mM NaCl, 5.9 mM KCl, 1.2 mM MgCl_2_, 11.6 mM HEPES (pH 7.3), 11.5 mM glucose and 1.5 mM CaCl_2_), followed by a de-esterification step of 30 min in the absence of Fura-2 AM. During the de-esterification step, cells were treated with vehicle, U73343 (1 and 2.5 µM) or U73122 (1 and 2.5 µM). Fluorescence was monitored on a luminescence spectrometer (AMINCO-Bowman Series 2, Spectronic Unicam) by alternately exciting the Ca^2+^ indicator at 340 and 380 nm and collecting emitted fluorescence at 510 nm. Basal [Ca^2+^]_cyt_ was derived after in situ calibration according to the Grynkiewicz equation: [49]

$$\left[ {\mathrm{{Ca}}^{2 + }} \right]_{\mathrm{{cyt}}}\left( {nM} \right) = K_d \times q \times \frac{{R - R_{{\it{min}}}}}{{R_{{\it{max}}} - R}}$$

*K*_d_ is the dissociation constant of Fura-2 for Ca^2+^ at room temperature (241 nM), *q* is the fluorescence ratio of the emission intensity in the absence of Ca^2+^ (F_380 max_), to that in the presence of saturating Ca^2+^ (F_380 min_), *R* is the fluorescence ratio, and *R*_min_ and *R*_max_ are the minimal and maximal fluorescence ratios, respectively. *R*_max_ was obtained by administrating 50 µM digitonin, subsequently *R*_min_ was measured by adding 33 mM EGTA in Ca^2+^-free modified Krebs solution.

### Ca^2+^ measurements in cell populations

To perform Ca^2+^ measurements in intact cells, DLBCL cells were seeded in poly-L-lysine-coated 96-well plates (Greiner) at a density of 5 × 10^5^ cells/ml. The cells were loaded for 30 min with 1.25 µM Fura-2 AM at 25 °C in modified Krebs solution, followed by a 30 min de-esterification step in the absence of Fura-2 AM. Fluorescence was monitored on a FlexStation 3 microplate reader (Molecular Devices, Sunnyvale, CA, USA) by alternately exciting the Ca^2+^ indicator at 340 and 380 nm and collecting emitted fluorescence at 510 nm, as described previously [50]. All data were obtained in triplicate and are plotted as F_340_/F_380_. At least three independent experiments were performed.

### Single-cell Ca^2+^ imaging

The IP_3_ sponge and mCherry constructs were introduced into SU-DHL-4 cells as described above. A Zeiss Axio Observer Z1 Inverted Microscope equipped with a 20x air objective and a high-speed digital camera (Axiocam Hsm, Zeiss, Jena, Germany) were used for these measurements. Fura-2 AM measurements were performed as previously described [15].

### Statistical analysis

Results are expressed as average ± SD or SEM as indicated. The number of independent experiments is always indicated. Significance was determined using a one-tailed or two-tailed paired Student’s *t*-test or an analysis of variance (ANOVA) as appropriate. Differences were considered significant at *P* < 0.05.

## Electronic supplementary material

Supplemental Table 1

Supplemental Figure 1

Supplemental Figure 2

Supplemental Figure 3
