# Supplementary material for: Constitutive IP3 signaling underlies the sensitivity of B-cell cancers to the Bcl-2/IP3 receptor disruptor BIRD-2
Source: Cell Death Differ. 2018 Jun 13;26(3):531–47. doi: 10.1038/s41418-018-0142-3 (PMC6370760; doi:10.1038/s41418-018-0142-3)
Supplement: Supplementary file 1 — Supplemental Table 1 [file 41418_2018_142_MOESM1_ESM.docx]

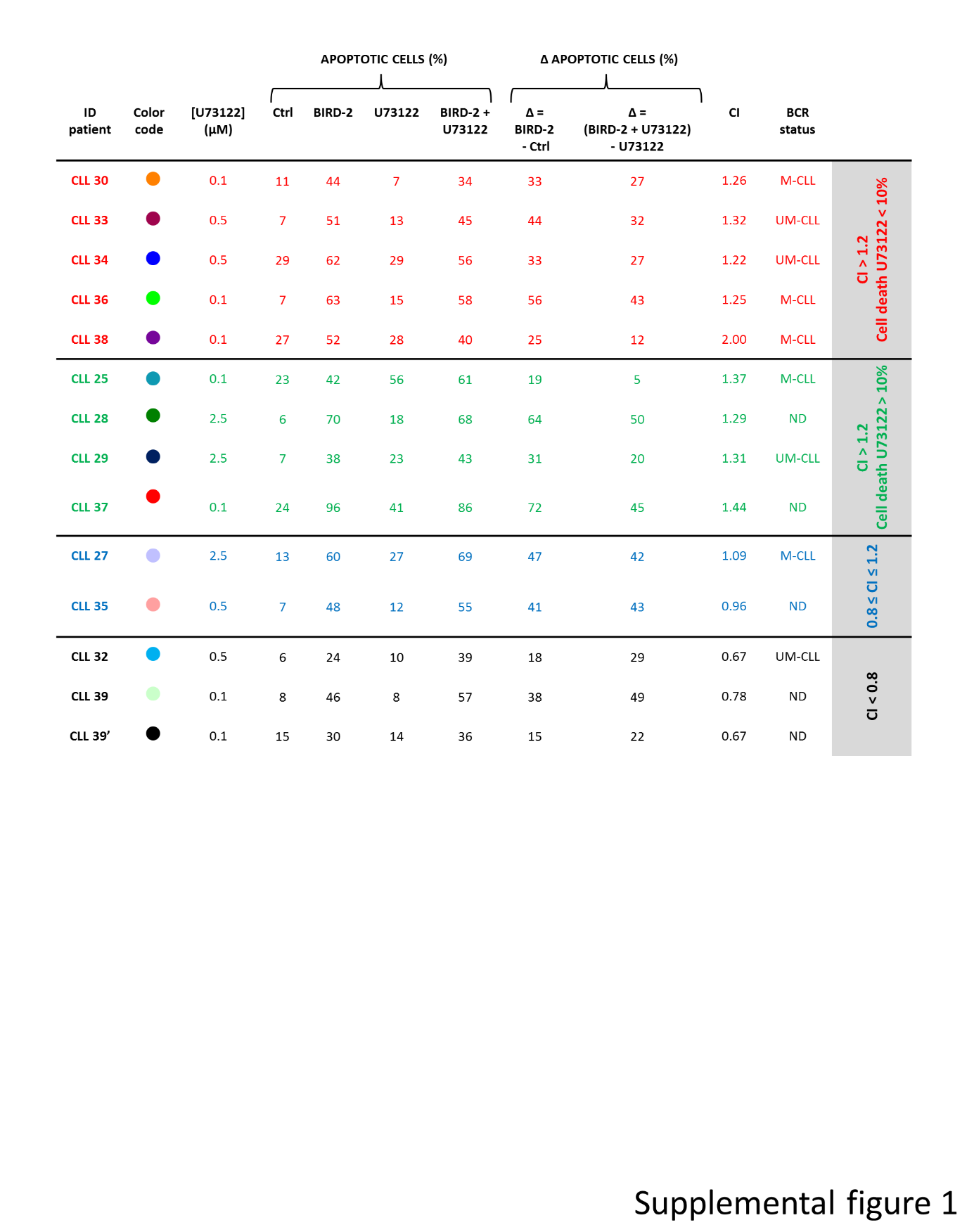


Supplemental Table 1 **Pharmacological PLC inhibition suppresses BIRD-2-induced apoptosis in a subset of primary CLL cells.** Table summarizing the data obtained for the primary CLL cells. Each CLL sample is represented with a different color. The lowest U73122 concentration for which an effect could be observed was used (0.1/0.5/2.5 µM). The percentage of apoptotic cells (%) is shown for untreated CLL cells (Ctrl) and cells treated with 30 µM BIRD-2, U73122 or BIRD-2 + U73122. To assess whether U73122 protected against BIRD-2-induced cell death the difference in apoptotic fraction (∆ apoptotic cells) between the BIRD-2-treated and the control condition, and between the BIRD-2 + U73122-treated and the U73122-treated condition was compared. The CI was calculated for the combined treatment of U73122 with BIRD-2. CLL cells were stratified into 4 different groups according to the CI and U73122-induced cell death: CI > 1.2 & cell death U73122 < 10% (red), CI > 1.2 & cell death U73122 > 10% (green), 0.8 ≤ CI ≤ 1.2 (blue), and 0.8 < CI (black). The immunoglobulin heavy-chain variable region (IGHV) mutational status of the CLL samples is indicated as well: M-CLL represents patients carrying mutated IGHV, UM-CLL patients with unmutated IGHV, while ND indicates that the BCR status has not been determined.
