# Supplementary material for: Constitutive IP3 signaling underlies the sensitivity of B-cell cancers to the Bcl-2/IP3 receptor disruptor BIRD-2
Source: Cell Death Differ. 2018 Jun 13;26(3):531–47. doi: 10.1038/s41418-018-0142-3 (PMC6370760; doi:10.1038/s41418-018-0142-3)
Supplement: Supplementary file 2 — Supplemental Figure 1 [file 41418_2018_142_MOESM2_ESM.docx]

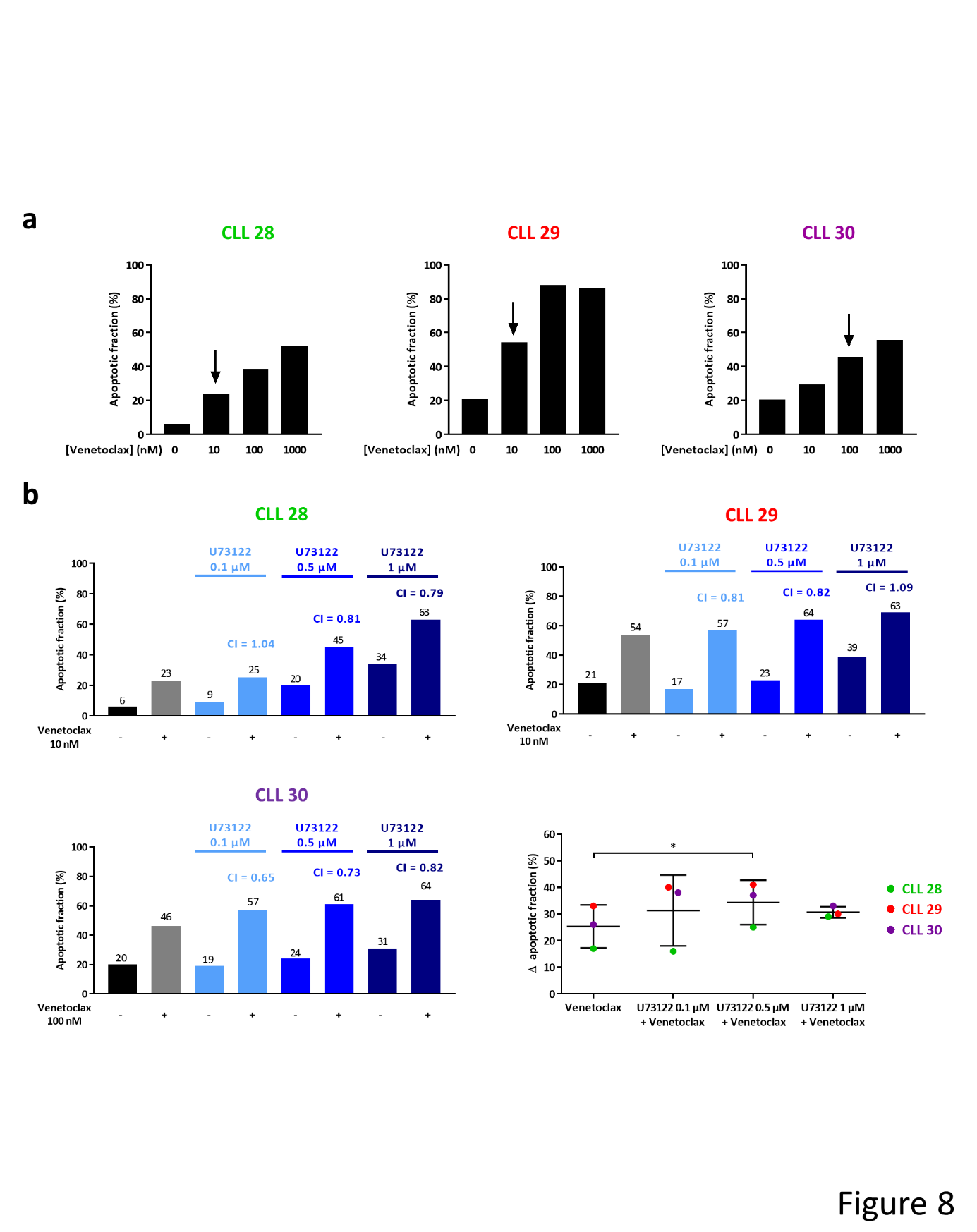


Supplemental Figure 1 **PLC inhibition does not protect against venetoclax-induced cell death in CLL cells.** (a) Apoptotic cell death measured in three CLL patient samples (CLL 28, CLL 29 and CLL 30) treated for 4 h with increasing concentrations of venetoclax (10 nM, 100 nM, 1 µM). A venetoclax concentration capable of inducing ~20 to ~30% cell death was chosen for further analysis, and this concentration is indicated by the arrow. (b) The apoptotic fraction measured in untreated CLL cells or cells treated for 4 h with venetoclax, U73122 or a combination of U73122 and venetoclax. Increasing concentrations of U73122 (0.1, 0.5, and 1 µM) were used. The combination index (CI) was calculated for each condition and is indicated in the panels. In the last panel, the ∆ apoptotic fraction (%), which was calculated in exactly the same manner as for BIRD-2 (see Fig. 7) by taking the difference in apoptotic fraction between the venetoclax-treated and the control condition, and between the venetoclax + U73122-treated and the U73122-treated conditions, is shown for the three CLL patient samples. Statistically significant differences were determined using an analysis of variance (ANOVA, * P < 0.05).
