# Supplementary material for: Constitutive IP3 signaling underlies the sensitivity of B-cell cancers to the Bcl-2/IP3 receptor disruptor BIRD-2
Source: Cell Death Differ. 2018 Jun 13;26(3):531–47. doi: 10.1038/s41418-018-0142-3 (PMC6370760; doi:10.1038/s41418-018-0142-3)
Supplement: Supplementary file 3 — Supplemental Figure 2 [file 41418_2018_142_MOESM3_ESM.docx]

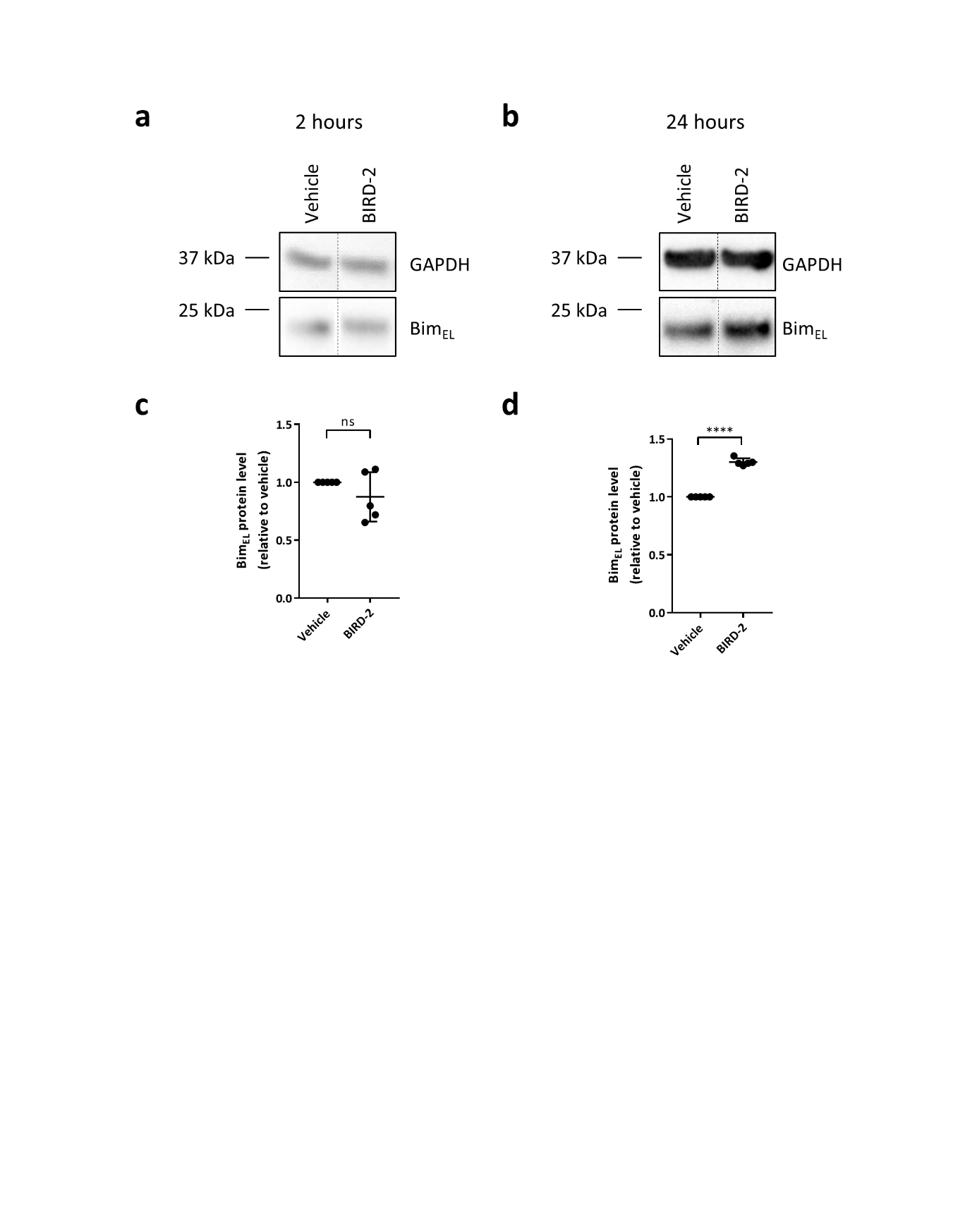
Supplemental Figure 2 **Expression of Bim is upregulated by BIRD-2 treatment in SU-DHL-4.** Expression levels of Bim_EL_, the most abundant isoform of Bim, detected in SU-DHL-4 cells treated for 2 (a) and 24 h (b) with BIRD-2 (10 µM). The expression level of GAPDH was used as a control for equal loading. The dotted lines on the blots indicate that the protein bands of the two conditions were not positioned side by side. However, the bands are from the same gel and they were obtained with the same exposure time. Moreover, we performed no change in contrast. The western blot is representative for 5 independent experiments. (c, d) Quantification of the Bim_EL_/GAPDH protein levels, relative to the level in vehicle-treated cells, which was set at 1. The results obtained after 2 h of treatment are shown in (c), whereas quantification of the results obtained after 24 h of treatment are shown in (d). Data are represented as the average ± SEM of 5 independent experiments. Statistically significant differences were determined using a two tailed paired t-test (**** P <0.0001).
