# Supplementary material for: Constitutive IP3 signaling underlies the sensitivity of B-cell cancers to the Bcl-2/IP3 receptor disruptor BIRD-2
Source: Cell Death Differ. 2018 Jun 13;26(3):531–47. doi: 10.1038/s41418-018-0142-3 (PMC6370760; doi:10.1038/s41418-018-0142-3)
Supplement: Supplementary file 4 — Supplemental Figure 3 [file 41418_2018_142_MOESM4_ESM.docx]

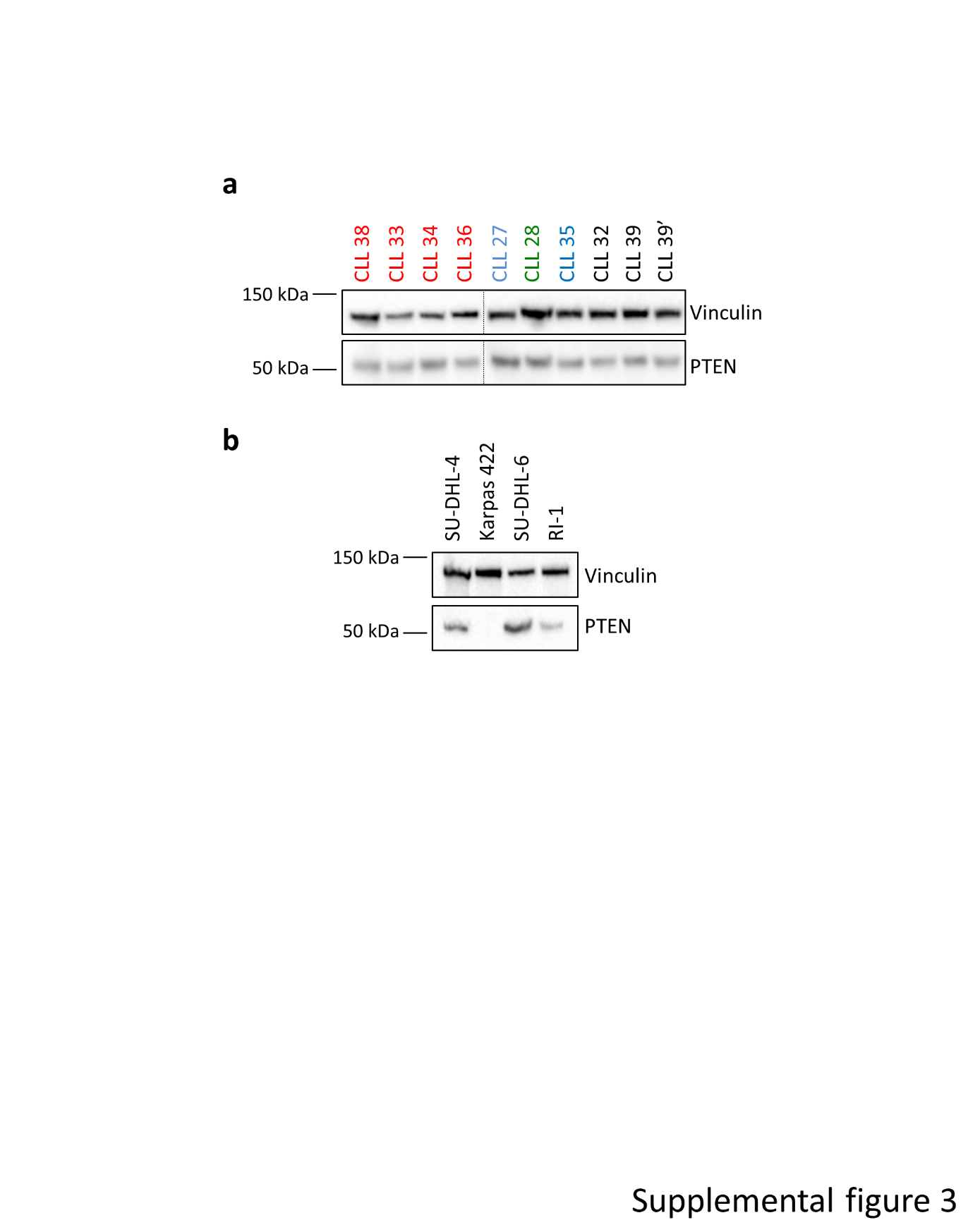


Supplemental Figure 3 **PTEN expression in primary CLL cells and DL-BCL cells lines.** (a) Expression levels of PTEN detected in CLL patient samples, which were stratified according to cell death triggered by U73122 and the CI calculated for U73122 + BIRD-2 treatment: CI > 1.2 & cell death U73122 < 10% (red label), CI > 1.2 & cell death U73122 > 10% (green label), 0.8 ≤ CI ≤ 1.2 (blue label) and CI < 0.8 (black label). (b) PTEN expression levels in DL-BCL cells. The expression level of vinculin was used as a loading control in both panels. The western blot is representative for 3 independent experiments.
